# Effect of Chromium Carbide Addition on the Microstructures and Properties in Dual Carbide Phases Reinforced Ni-Based Composite Coatings by Plasma Cladding

**DOI:** 10.3390/ma16134580

**Published:** 2023-06-25

**Authors:** Zhanji Geng, Mengling Zhang, Jianyong Zhu, Yingbo Peng, Wei Zhang, Feng Liu

**Affiliations:** 1 Powder Metallurgy Research Institute, Central South University, Changsha 410083, China; 2 Changsha Huaxi New Material Co., Ltd., Changsha 410083, China; 3 Hunan Hastion Technology Co., Ltd., Zhuzhou 412000, China; 4 Hunan Metallurgy Material Institute Co., Ltd., Changsha 410129, China; 5 College of Engineering, Nanjing Agricultural University, Nanjing 210031, China

**Keywords:** chromium carbide, microstructure, wear resistance, plasma cladding, metal-based titanium carbide composite coating

## Abstract

Cr_3_C_2_-modified NiCr–TiC composite coatings were prepared using the plasma spraying technique for different Cr_3_C_2_ contents on the microstructure and the properties of the Ni-based TiC cladding layer were investigated. The microstructures of the coatings were characterized using scanning electron microscopy, and the friction and wear performance of the coating was evaluated by the wear tests. The results revealed that the surfaces of the Cr_3_C_2_-modified NiCr–TiC composite coatings with varying Cr_3_C_2_ contents were dense and smooth. TiC was uniformly distributed throughout the entire coating, forming a gradient interface between the binder phase of the Ni-based alloy and the hard phase of TiC. At high temperatures, Cr_3_C_2_ decomposes, with some chromium diffusing and forming complex carbides around TiC, some chromium solubilizes with Fe, Ni, and other elements. An increase in chromium carbide content leads to an upward trend in hardness. The measured hardness of the coatings ranged from 600 to 850 HV3 and tended to increase with increasing Cr_3_C_2_ content. When the mass fraction of Cr_3_C_2_ reached 30%, the hardness increased to 850 HV3, and the cracks and defects were observed in the coating, resulting in a wear resistance decline.

## 1. Introduction

Particle-reinforced metal matrix composites significantly enhance the wear resistance of coatings. By leveraging the advantageous physical, chemical, and mechanical properties of the reinforcing materials, these coatings compensate for the deficiencies of the substrate material, resulting in a superior overall performance. These coatings are mainly applied in industries requiring surfaces with excellent wear resistance and components with long lifespans, such as in the engineering of mixed-material mixing drums for new energy equipment, marine engineering, and automotive turbochargers [1,2]. The type and amount of the reinforcing phase added to the composite determines the operating performance of the coating.

Typically, the reinforcing phases used in composite coatings are metal carbides such as WC, TiC, TiN, and CrC. The addition of carbides can be achieved through ex situ and in situ methods. In situ generated carbides tend to be uniformly distributed and well bonded to the matrix; however, their content is remarkably limited, restricting the potential for improvements in hardness and wear resistance. For example, composite coatings with different TiC/Co50 contents were produced using a laser-cladding technique [3]. As the TiC content increased, the hardness of the cladding layer increased, reaching approximately four-fold that of the substrate. The best overall performance of the cladding layer was achieved when the TiC content reached 20%. Sun Shuting [4] used laser cladding to prepare NbC-reinforced nickel-based composite coatings in situ and determined that the optimal content was 20% based on the coating’s microstructure, hardness, and wear resistance. Ex situ addition of TiC can significantly increase the carbide content and improve wear resistance. Yi, X.X, et al. [5] improved the content and formation rate of NbC by appropriate ex situ addition while inhibiting the formation of the eutectic structure and promoting the formation of blocky and petal-like carbides. However, the wetting behavior between the matrix and carbides greatly affects the size and content of carbides. The uniform distribution of a high-volume fraction of hard and brittle carbide particles is difficult to control, and the agglomeration of hard particles on the metal matrix can lead to stress concentration, crack formation, and formation of micropores and other defects, which can lower the toughness of the composite coating and result in cracking and delamination. In particular, titanium carbide, which has superior wear resistance, higher hardness, and lower cost compared with tungsten carbide, is currently being extensively investigated for different applications. However, the poor wetting behavior of titanium carbide with iron-group elements affects its application [6,7,8,9,10].

Nickel-based self-fluxing alloy powder is the most commonly used commercial matrix material [11]. It has good wetting behavior with TiC, resulting in a strong interfacial bond, and exhibits high temperature and corrosion resistance. Furthermore, it is less expensive than cobalt and has a better high-temperature performance than iron-based materials. Nickel also has excellent wettability with TiC and easily penetrates the interior of the particle skeleton. Nickel-based TiC composite coatings are used in wear-resistant and high-temperature applications [4]. However, owing to the incomplete wetting of nickel by TiC, the performance of the composite coating is limited by the amount of titanium carbide added. Excessive TiC particles tend to agglomerate and grow, and TiC particles can undergo decarburization and transform into Ti_8_C_5_ at high temperatures. The decomposed carbon can then diffuse into the matrix, causing changes in the matrix structure, including the formation of brittle structures such as martensite and ledeburite [12]. This can result in cracks in the matrix and coating during the cladding or cooling process, leading to coating defects and insufficient performance. It has been shown that the optimal TiC content for laser cladding is approximately 30% [13], whereas it is approximately 10–20% for plasma cladding [14,15,16]. 

In the WC/Ni-based composite coating, Lifeng et al. [17] added 20% TiC, resulting in the formation of dispersed TiWC_2_ in the cladding layer. The morphology of the reinforcing phase changed; the sharp parts smoothened, and the amount of white fishbone-like structure increased, further enhancing the wear resistance of the composite coating. Shuting et al. [4] added TiC to the NbC/Ni-based composite coating, as their lattice structures are similar. During the cladding process, the two lattices easily combine and grow into (Ti, Nb)C, which can stably exist and further increase the hardness. The Cr_3_C_2_/Ni-based composite coating with added TiC exhibits excellent high-temperature and corrosion-resistance properties [18]. In the TiC/Ni coating prepared by Chengcai et al. [19], the addition of chromium (Cr) results in the formation of a solid solution during the laser-cladding process, as Cr, Ti, and C generated from the decomposition of TiC particles dissolve into the Ni matrix, leading to solid solution strengthening. According to the Cr–C phase diagram [20,21,22], the chromium carbide (CrC) phase appears at higher temperatures, which can decompose into Cr_3_C_2_ and graphite. At lower temperatures, it can also dissolve with titanium carbide, achieving stabilization. Therefore, the addition of multiphase carbides can further improve the coating performance and mitigate the reduction in the toughness of traditional composite coatings caused by the addition of a high-volume fraction of a single reinforcing phase.

In this study, TiC-rich composite coatings were prepared using plasma cladding and the addition of Cr_3_C_2_, which significantly improved the wear resistance of the coating. The effect of the Cr_3_C_2_ content on the phase composition, microstructural evolution, friction, and wear performance of the coatings was evaluated. These findings provide theoretical and experimental evidence that can be used to enhance the performance and expand the application scope of particle-reinforced metal matrix composite coatings.

## 2. Materials and Methods

### 2.1. Materials 

The base material used in the experiment was high-carbon steel, with sample dimensions of 150 mm × 150 mm × 15 mm. The cladding material consisted of Ni25A alloy powder (Jiangxi YuTech Powder Metallurgy Co., Ltd., Pingxiang, China) (composition shown in Table 1) with a particle size range of 20–50 μm and TiC powder (Hunan Hastion Technology Co., Ltd., Zhuzhou, China) with a particle size range of 50–150 μm, which was prepared by a carbothermic reduction and screened to the desired size range. Spherical Cr_3_C_2_–25% NiCr powder (Jiangxi YuTech Powder Metallurgy Co., Ltd.) was produced by granulation using vacuum spray granulation equipment, followed by vacuum sintering for shaping. The TiC mass fraction was 45%, and varying contents of Cr_3_C_2_–25% NiCr (20, 25, 30, 35, and 40%) were added; accordingly, the mass fractions of Cr_3_C_2_ were 15, 19, 23, 26, and 30%, respectively (composition shown in Table 2).

### 2.2. Methods

The composite powders were mixed using an ultrasonic vibration mixer for 1 h. The cladding experiments were conducted using Duomu A300 plasma cladding equipment. During the cladding process, a coaxial carrier gas powder feeding method was used for single-layer multiple-pass cladding, with argon gas protection. The parameters are presented in Table 3. The test samples were polished by the ZXQ-5HS inlay machine gold sandpaper (60 #to2000#), and then on the MP-3S-2 with 15 micron, 8 micron, 1 micron automatic polishing machine, each polishing time is 3 min. Phase analysis of the cladding layers was performed using a Bruker D8 X-ray diffractometer. The parameters were as follows: operating voltage of 40 kV, operating current of 40 mA, Cu Kα radiation, step scan speed of 2°/min, and scanning range of 20~90°. Microstructural observation and analysis of the cladding layers were carried out using a Tescan Mira4 field-emission scanning electron microscope. The microhardness of different positions along the cross-section of the laser cladding layer at the same horizontal level was measured using a Huayin 200HV-5 Vickers hardness tester. A five-point average was taken, with a test load of 3000 g (30 N) applied for 10 s. The friction and wear performance of the cladding layers were evaluated using an MM-U10G pin-on-disk tribometer at room temperature with a load of 50 N, rotational speed of 50 r/min, and wear time of 1 h, using a silicon nitride ball with a diameter of φ 9.425 mm as the counterface. After the wear test, the mass loss owing to friction was determined using a FA2004 electronic balance with a precision of 0.1 mg.

## 3. Results and Discussion

### 3.1. Phase Structure of the Cladding Layer

**Figure 1:**
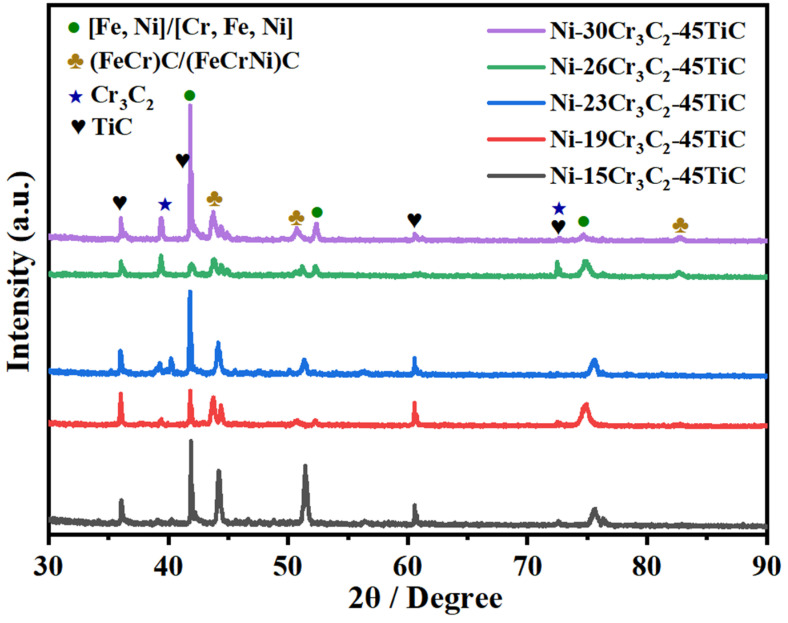
XRD pattern of composite coating.

Figure 1 shows the X-ray diffraction (XRD) pattern of the composite coating. The results indicate that the microstructure of the cladding layer is composed of solid solution phases such as FeNi/NiCrFe, TiC, (Fe, Cr)C/(Fe, Cr, Ni)C, Cr_7_C_3_, and complex carbides. According to the literature [23], the enthalpy of formation (ΔrH) for Cr_3_C_2_ is –0.114 eV/atom. The enthalpies of formation for the solid solution phases were calculated using Materials Studio software (Version 8.0); the enthalpies for (NiCr_11_)C_8_, (Ni_2_Cr_10_)C_8_, and (Ni_3_Cr_9_)C_8_ were calculated to be −0.124, −0.0758, and +0.1298 eV/atom. This indicates that when Ni ≥ 3, the enthalpy of formation for the substituted solid solution phases becomes positive, and thus solid solution phases cannot be formed. Cr_3_C_2_ decarburizes at high temperatures to produce Cr_7_C_3_. The dissolution of chromium carbides into TiC causes changes in the lattice parameters of the δ phase. Wenlong et al. [20] found that when the chromium carbide content was TiC, TiC–6.7% Cr_3_C_2,_ TiC–19.5% Cr_3_C_2_, and TiC–30% Cr_3_C_2_, respectively, the lattice parameters changed at 1500 °C to 4.33^−10^, 4.31^−10^, 4.27^−10^, 4.25^−10^ m, which remain essentially unchanged. Moreover, for the Cr_3_C_2_ solid solution in TiC, a strong and stable structure was formed.

During the plasma cladding of nickel-based carbide composite coatings on an iron base plate, melting of the matrix provides the Fe source, and Cr_3_C_2_ pyrolysis provides the Cr and C sources. Under the high-temperature conditions of plasma cladding, a compound FeNi/NiCrFe and (FeNi)C/(FeCrNi)C solid solution was produced, with a significant peak value [24,25,26,27,28]. With increasing Cr_3_C_2_ content, the main phase types of the different composite coatings did not change. They were composed of TiC, Cr_3_C_2_, FeNi/NiCrFe, (FeNi)C/(FeCrNi)C solid solution and other phases. The main diffraction peaks of each phase were enhanced and sharpened to varying degrees; however, the positions of the diffraction peaks did not shift. The XRD spectrum revealed that, owing to the role of the high-energy plasma beam, Cr_3_C_2_ added to the nickel-based alloy undergoes a large amount of decomposition, which significantly increases the Cr and C contents in the culture pool. During the rapid solidification process, a large number of solid solutions and carbides will be generated, followed by the formation of (FeNi)C/(FeCrNi)C solid solution.

### 3.2. Morphology of the Cladding Layer

**Figure 2:**
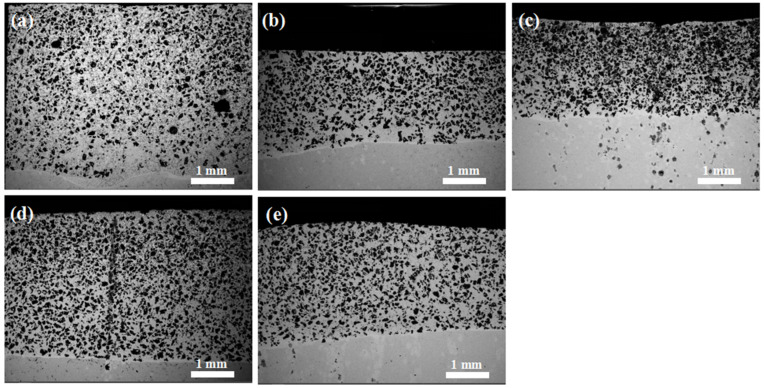
Cross-sectional SEM images of the composite coatings with (**a**) 15%, (**b**) 19%, (**c**) 23%, (**d**) 26%, and (**e**) 30% Cr_3_C_2_.

Figure 2 shows SEM images of the polished cross-sections of the NiCr-TiC-based composite coatings with different nominal Cr_3_C_2_ contents (as listed in Table 2). The coatings are designated 1# for the Ni-15Cr_3_C_2_-45TiC coating with 15% Cr_3_C_2_ content, 2# for the Ni-19Cr_3_C_2_-45TiC coating with 19% Cr_3_C_2_ content, 3# for the Ni-23Cr_3_C_2_-45TiC coating with 23% Cr_3_C_2_ content, 4# for the Ni-26Cr_3_C_2_-45TiC coating with 26% Cr_3_C_2_ content, and 5# for the Ni-30Cr_3_C_2_-45TiC coating with 30% Cr_3_C_2_ content, respectively. Figure 2 shows that the cladding layer can be divided into three distinct regions: the heat-affected zone, the bonding zone, and the cladding zone. The bonding zone appears as a prominent “bright” band, which was formed by the mutual dilution of the substrate metal and the cladding powder during the plasma cladding. The bonding zone acts as a transition region between the substrate material and coating, indicating a metallurgical bond between them.

### 3.3. Microstructure of the Cladding Layer

**Figure 3:**
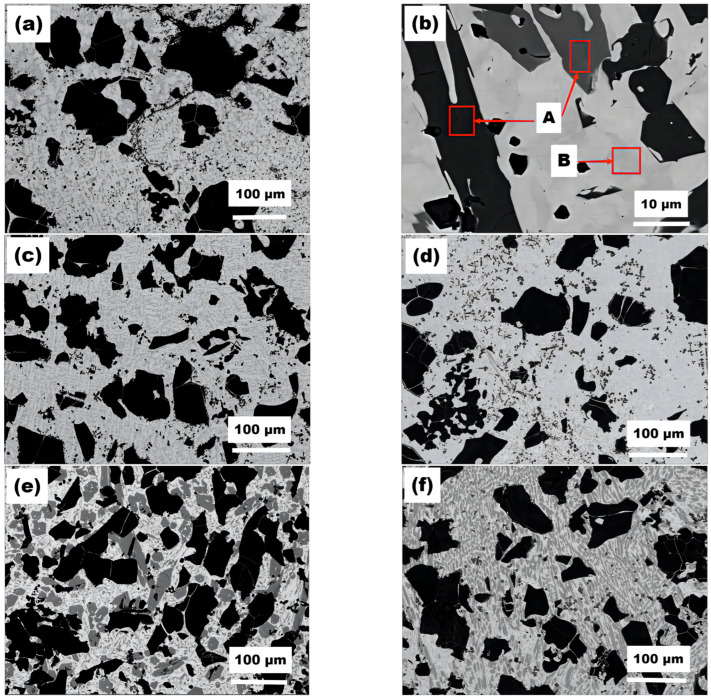

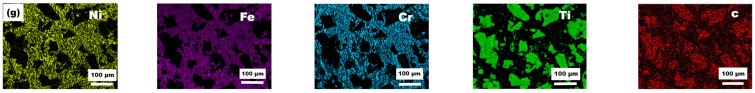
Morphology of the cladding layers: (**a**) low- and (**b**) high-magnification SEM images of coating 1#; low-magnification SEM images of coating (**c**) 2#, (**d**) 3#, (**e**) 4#, and (**f**) 5#; and (**g**) low-magnification elemental maps of coating 5#.

SEM images of the cross-sectional micromorphology of the plasma cladding layer with various mass fractions of Cr_3_C_2_ are presented in Figure 3. The black phase represents the TiC phase, which is uniformly distributed in the coating. In the coating with 15% Cr_3_C_2_, the second phase is present in smaller quantities and has smaller particle sizes, with a relatively uniform distribution. However, when the mass fraction of Cr_3_C_2_ is increased to 30%, the particle TiC size remains similar and relatively unchanged, whereas the size of the second phase significantly increases. With increasing distance from the bottom of the molten pool, the microstructure of the cladding layer transitions from cell grains to dendritic grains, and at the top of the cladding layer, it transforms into a fully disordered dendritic microstructure. This is likely influenced by the polycrystalline orientation of the base material and possible nucleation at the solidification front of the molten pool.

The microstructure of the cladding layer consists of a dendritic solid solution, eutectic structure, undissolved chromium carbides, as well as rod- and block-like carbide phases. As the Cr_3_C_2_ content increases, the dendritic grains in the cladding layer transform into equiaxed grains, resulting in a finer microstructure and a higher number of block-like carbide phases, as confirmed by XRD analysis. Additionally, a significant amount of undissolved titanium carbides are uniformly distributed in the coating, which can be attributed to the rapid cooling rate during the plasma fusing process.

**Figure 4:**
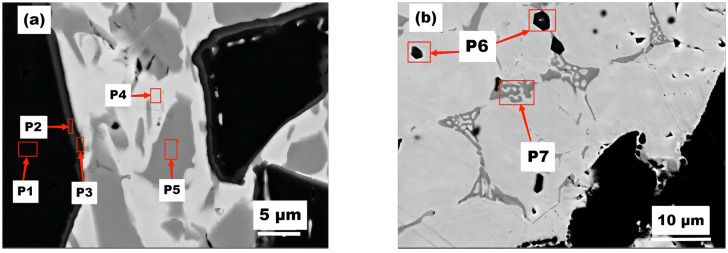
Different shape phase points (**a**) and (**b**) SEM image of coating 1#.

The high-magnification image of the 1# cladding layer, as shown in Figure 4, reveals the presence of numerous black TiC particles, as well as a significant amount of grey elongated and block-like structures. Energy-dispersive spectroscopy (EDS) analysis (Table 3) indicates that Point A contains a high concentration of Fe, Cr, and Ni, while Point B is primarily composed of Fe and Ni, with lower Cr content. Combined with the XRD results (Figure 2), it can be concluded that the phases at Point A are (Fe, Cr)C and (Fe, Cr, Ni)C, while Point B contains (Fe, Cr) and (Cr, Fe, Ni) solid solutions owing to the incorporation of Cr. The EDS results of the TiC region in the 1# cladding layer (Table 4) reveal that during the cladding process, a significant amount of iron from the substrate melted into the coating [29]. The contents of other elements may vary depending on the specific region selected for analysis.

Coating #1 was further using EDS, revealing the formation of multicomponent carbides (NixCryFezTih)C with a thickness of about 0.5–1 μm around the TiC phase. The values of x, y, z, and h gradually transitioned as the thickness increased, until the outermost titanium (Ti) disappeared completely. The results for P1–P5 (Table 5) show that the decreasing Ti content is accompanied by a further transition to multicomponent carbides, such as (FeNi)C and (FeCrNi)C occurs. The elemental distribution at point P4 indicates that the melted Cr_3_C_2_ provides sufficient carbon for the precipitation of a second phase, namely (Fe, Cr, Ni)C. The results from point P5 indicate the formation of an FeNi solid solution enabled by the high Fe and Ni contents in the substrate [30].

During the melting process, the melted TiC reacts with decomposed Cr_3_C_2_ and Fe to form ternary carbides, as shown in Table 4, Table 5 and Table 6, with precipitation of a second phase observed at Point P6. Meanwhile, the unmelted large TiC particles remain intact. At Point P7, a network-like binary carbide consisting of Fe and Cr is formed. With an increase in the Cr_3_C_2_ content, both the quantity and size of the formed complex carbides increase.

### 3.4. Mechanical Properties of the Cladding Layers

#### 3.4.1. Microhardness

**Figure 5:**
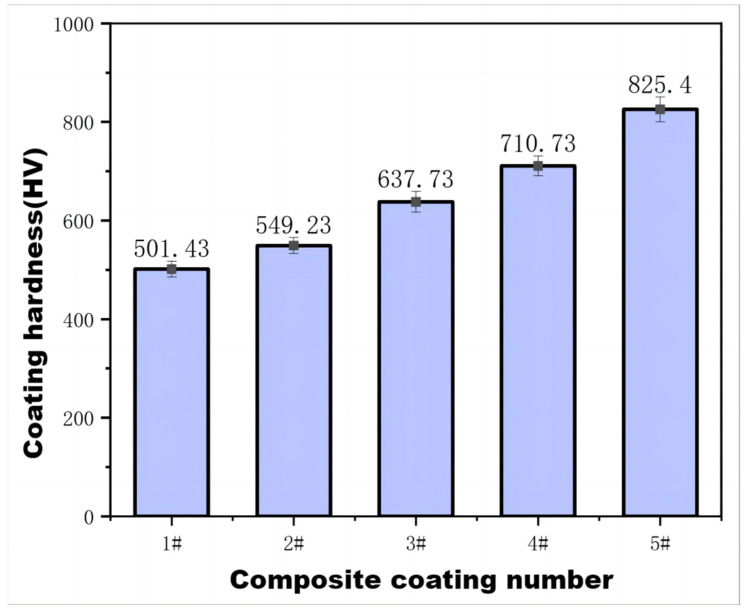
Hardness of composite coatings.

Pokhmurska et al. [31] showed that Cr_3_C_2_ dispersed in the solid solution can effectively enhance the hardness of the coating. Figure 5 compares the average surface and cross-sectional hardness values of the cladding layer, revealing that the hardness of the composite coating generally increases with the increase in Cr_3_C_2_ content, reaching a maximum of 825.4 HV3. 

The analysis results indicate that the morphology of the TiC particles in the coating remains largely unchanged, while the primary changes occur in the binder-phase nickel-based alloy, Cr_3_C_2_, and in the melted Fe. Owing to the low melting point of Cr_3_C_2_, it dissolves in the molten pool under the action of a high-temperature plasma arc, providing a significant source of Cr and C. For one thing, Cr and C are supersaturated in the solid solution, enhancing the solid-solution strengthening effect of the alloying elements. For another thing, during the cooling process, they can further react with C, B, and other elements to form more compounds, thus further improving the performance of the coating. Partial melting of Cr_3_C_2_ and subsequent cooling results in the precipitation of Cr_3_C_2_ particles as non-uniform nuclei that promote nucleation and refining of the microstructure of the coating, thus providing a grain refinement strengthening effect. In addition, the area surrounding the black TiC particles is mainly composed of (NiCrFe)C and (FeCr)C along with dispersed fine carbides such as TiC. Among them, the γ-Ni dendritic solid solution, which contains dissolved C, Cr, Si, Fe, and other elements, possesses a eutectic structure with compound phases. Based on the morphology and X-ray analysis of the coating, the region with higher Cr content is considered the primary Cr-rich phase and is believed to contain a certain amount of dissolved Ni, Fe, and other elements. During the solidification process, the harndess of the cladding layer is enhanced via solid solution, dispersion, and grain refinement effects. The composite coating contains a significant amount of TiC and hard phases of complex carbides, which serve as the supporting framework of the entire cladding layer, further improving the hardness of the coating [32].

#### 3.4.2. Nanoindentation

**Figure 6:**
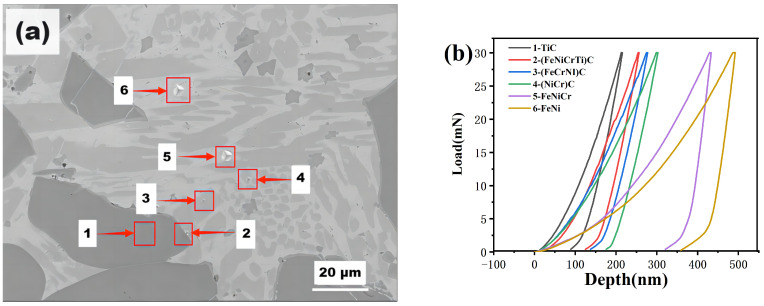
(**a**) Indentation points within different phases and (**b**) load-depth curves for coating 4#.

Nanoindentation testing was performed on sample 4# (Figure 6). Based on the results of the XRD and SEM–EDS analysis, Point 1 can be concluded to correspond to TiC particles; points 2–4 correspond to complex carbides; and Points 5 and 6 correspond to NiFe/FeNi solid solution. The microhardness results are shown in Table 7.

The large TiC particles exhibit a high hardness of 32 GPa. The (TiCr)C/(TiCrFe)C carbides have hardness values exceeding 20 GPa, indicating high hardness. The NiFe/FeNi solid solution exhibits a hardness above 6 GPa. This further confirms that Cr_3_C_2_ dissolves significantly during the high-temperature process of thermal spraying, forming multicomponent carbides around the TiC particles, including (TiCr)C/(TiCrFe)C multicomponent carbides with other elements. With increasing Cr_3_C_2_ content, the volume fraction of multicomponent carbides in the coating increases, which enhances the hardness and elastic modulus. The NiFe and FeNi phases exhibit high hardness and toughness. The high-volume fraction of hard phases helps to reduce thermal stresses and improve the coating’s defect structure, thereby enhancing the overall performance of the coating.

#### 3.4.3. Analysis of Friction and Wear Performance of the Composite Coating

**Figure 7:**
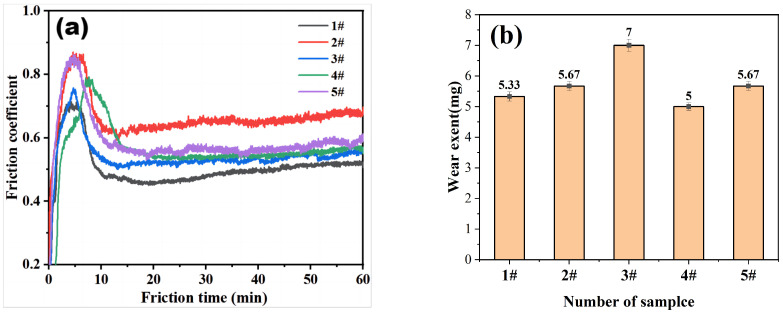
(**a**) Friction coefficient and (**b**) wear weight loss of the composite coating.

Figure 7 compares the friction coefficient and weight loss due to sliding wear for composite coatings with different Cr_3_C_2_ contents. The coating with 26% Cr_3_C_2_ addition exhibits a lower friction coefficient and the least amount of wear. Although the coating labeled 1# has a lower friction coefficient, it also has a lower hardness, resulting in a higher weight loss compared to coating 4#. 

#### 3.4.4. Wear Morphology

**Figure 8:**
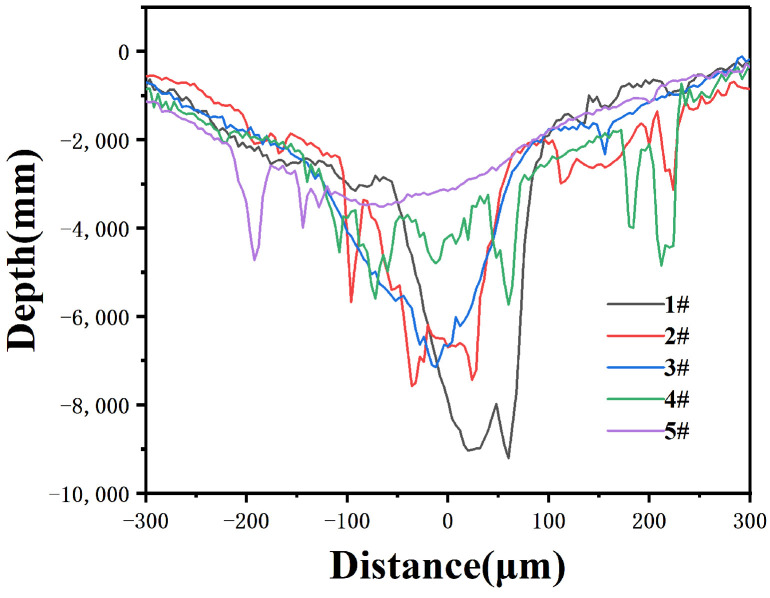
Cross-sectional profile of the composite coatings.

Figure 8 shows the cross-section of the composite coating after friction testing. The wear coefficients of the samples are shown in Table 8. The 1# coating exhibits a lower friction coefficient, but larger wear dimensions in terms of the width and depth of the wear marks. This is mainly due to its lower hardness, resulting in higher wear compared to the 4# coating. The friction coefficient of sample 4# was lower than that of sample 5#. Although the hardness of sample 4# was lower than that of sample 5#, the width and depth of the wear marks were smaller in sample 4#, indicating excellent wear resistance performance. For sample 5#, the width and depth of the wear marks were measured to be 924.43 μm and 35 μm, respectively. For sample 4#, the width and depth were measured to be 845.23 μm and 8 μm, respectively.

During friction and wear testing, the friction coefficient curve was directly obtained using software (origin2021), and the wear rate (WR; mm^3^/N·m) was calculated using Equation (1).

WR = V/FL(1)

Here, V is the wear volume (mm^3^), F denotes the normal force applied during the wear process (in N), L represents the total friction stroke (m), and WR represents the wear rate (mm^3^/N·m), Therefore, WR indicates the wear volume per unit wear distance under a unit load.

- Table data in Table 8 indicate that the wear resistance of the composite coating is exceptional. This improvement in wear performance aligns with Archard’s wear theory [13], which suggests that the wear volume (Vw) is directly proportional to the wear load, but inversely proportional to the material’s hardness (Figure 5). The composite coating exhibits higher hardness and thus superior wear resistance. Additionally, the lower friction coefficients contribute to further the wear performance of these coatings.

**Figure 9:**
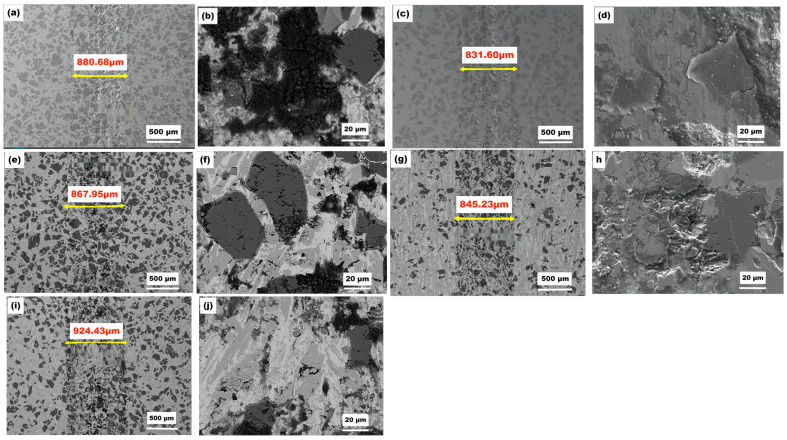
Wear marks on the coatings with various Cr_2_C_3_ contents. (**a**,**b**) 1#, (**c**,**d**) 2#, (**e**,**f**) 3#, (**g**,**h**) 4#, and (**i**,**j**) 5#.

Figure 9 shows that the widths of the wear marks on coatings 2# and 4# are remarkably small. In terms of friction coefficient and wear volume, sample 4# exhibits the best wear resistance. Microscopically, numerous branching cracks can be observed on the surface of the hard phase. This may be due to crack deflection around the two-phase particles and the bending of crack fronts between particles, resulting in the reduced driving force for crack propagation and increased fracture toughness. The plastic deformation zone ahead of the crack absorbs some energy, hindering crack propagation [33,34,35].

The wear surface contains hard phases of TiC, (FeCr)C, (FeCrNi)C, FeNi, FeNiCr, oxide, and bonded Si_3_N_4_, among others. Scanning electron microscopy (Table 9) reveals that the wear mechanism of the samples is abrasive wear, with some accumulation of wear debris during the wear process. This accumulation of wear debris increases the roughness of the wear surface, which is one of the main reasons for the relatively high friction coefficient of the material under room temperature conditions.

**Figure 10:**
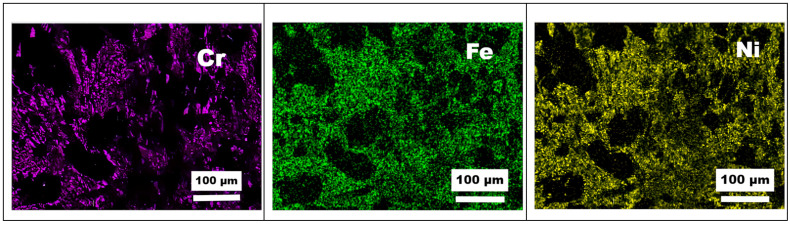

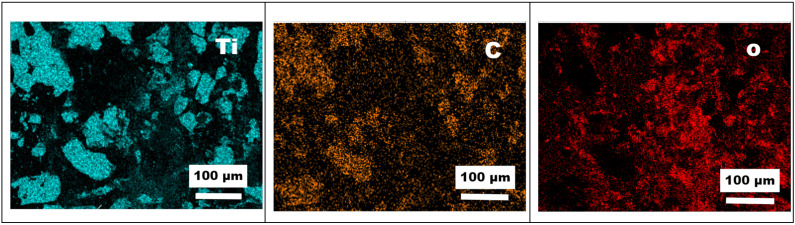
SEM–EDS images of the wear mark morphology of coating 1#.

The micromorphology and energy spectrum chemical composition analysis of the wear scars after the wear test of the composite coating are shown in Figure 9 and Figure 10, respectively. The composite coatings exhibit adhesive wear characteristics, with the formation of thick oxide films on the friction surfaces [36,37]. The morphology of the wear debris at room temperature is shown in Figure 9. The energy spectrum reveals that the white particles are mainly composed of titanium carbide, chromium carbide ceramic particles, FeCrNi, and some SiN dual-phase debris. This is primarily because, during the wear process, ceramic phases fracture and accumulate on the surface, forming nanosized ceramic particles after repeated rolling and fragmentation by the abrasive balls. The gray portion consists of a mixture of Cr_3_O_2_, FeO, and NiO oxides. In addition, the energy spectrum indicates that the upper oxide layer is mainly composed of Ti, Cr, and Ni oxides, while the lower oxide layer is primarily composed of Ti oxides. Similar results are observed in the wear scars of the composite coating. These oxides are formed owing to the high temperature generated during the friction process, resulting in rapid surface oxidation and effectively improving the wear resistance of the coating. The Gibbs free energy diagrams of the elemental oxidation reactions reveal that the oxidation of TiC into TiO_2_ and CO_2_ has the lowest energy compared with the other oxidation reactions [38]. This suggests that TiC preferentially forms oxides during the friction process and accounts for the significant amount of Ti oxide in the oxide films generated in the composite coating during friction. The formation of TiO_2_ and other metal oxides during the friction process is advantageous because they are relatively soft and possess lubricity [39]. This allows for the development of a continuous friction-induced oxide film, which exhibits lubricating properties. The presence of such a lubricating and continuous oxide film further enhances the wear resistance performance of the coating.

The abrasion debris exhibits larger delamination in a layered form. In Figure 9, it can be observed that under room temperature conditions, the composite coating predominantly undergoes abrasive wear with abrasive particles when in contact with a silicon nitride counterpart [40], and a minor amount of oxidative wear. Fracture of titanium carbide and multicomponent carbide particles occurs in the sub-surface region, with cracks primarily appearing at the interface between the multicomponent carbide particles and the metal binder phase.

**Figure 11:**
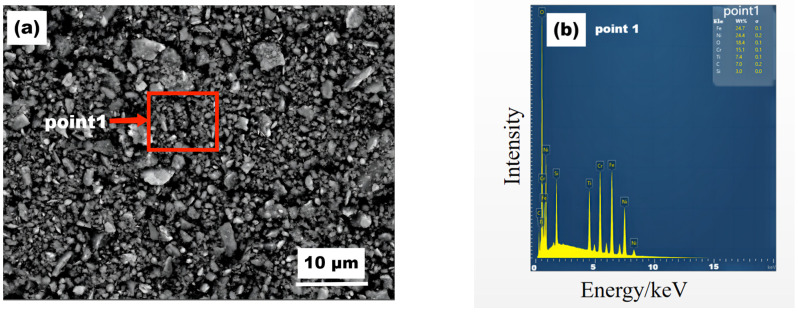
(**a**) SEM image and the (**b**) corresponding EDS spectrum of Point 1 in (**a**) of the coating debris on coating 4#.

SEM and EDS analysis were performed on the wear debris of coating 4#, as shown in Figure 11, respectively. The wear resistance of the composite coating is mainly attributed to the presence of titanium carbide (TiC), as revealed by EDS analysis of the wear debris. As the Cr_3_C_2_ content varies in the range of 15% to 26%, the increase in FeCr carbide (FeCr)C content gradually leads to higher friction coefficients, increased hardness, and improved wear resistance. However, when the Cr_3_C_2_ content exceeds 30%, the hardness increases, but cracks penetrating the coating appear, resulting in more coating defects and a decrease in wear resistance.

### 3.5. In Situ Formation of the Hard Phase

To explain the formation mechanism of FeNi/NiCrFe,(Ti,Cr)C and (FeNi)C/(FeCrNi)C, and their influence on the coating properties, the solidification process of the molten tank was considered. During the plasma cladding process, when the melting pool temperature exceeds 2000 °C, Cr_3_C_2_ is decomposed into CrC molecules to form a composite carbide transition layer around TiC and form a stable binding body with the matrix. The melting of Fe, Ni, Cr, and C is generated in situ (FeNi)C/(FeCrNi)C. The melting point of the nickel-based alloy was approximately 1300 °C, of TiC was approximately 3067 °C, and of Cr_3_C_2_ was approximately 1890 °C. Cr_3_C_2_ and TiC will preferentially nucleate during the pool solidification. The study showed that (Ti, Cr) C formed, even though the melting pool temperature was below the melting point of the nickel–iron alloy. According to the B–C–Cr ternary phase diagram, the melting point of Cr_3_C_2_ is higher than that of Cr_23_C_6_. The addition of Ti and Cr consumed some C atoms and increased the Cr/C ratio, favoring Cr_3_C_2_ formation in the B–C–Cr system, while Cr_23_C_6_ was inhibited. This was the primary reason for the presence of numerous stable carbides in the coating and improved wear resistance [41].

Compound carbide particles formed in situ have high hardness and are uniformly distributed in the coating, which improves the coating wear resistance (see Figure 6). Additional hot melting of Cr_3_C_2_ caused a sharp increase in the contents of carbide in (TiCr)C and (FeNi)C/(FeCrNi)C in the coating. The high hardness of the carbides in the coating can hinder the dislocation movement and deformation of the substrate and play a significant role in pinning the substrate. Thus, the strength of the substrate is enhanced, and the wear resistance of the coating is improved. In addition, Fe in the Fe matrix, Ni form Ni25, and Cr atoms form Cr_3_C_2_ form a solid reinforcement phase that increases the hardness of the matrix and improves the wear resistance of the coating.

## 4. Conclusions

1. Plasma-sprayed Ni-based TiC composite coatings with various Cr_3_C_2_ contents exhibit a smooth surface morphology, dense structure, high bonding strength, and high microhardness. The composite coatings are composed of TiC, (FeCr)C, and (Fe, Cr, Ni)C multicomponent carbides, as well as (FeCr) and (Fe, Cr, Ni) solid solutions.
2. As the mass fraction of Cr_3_C_2_ increases, the microhardness of the plasma-sprayed coating also tends to increase. When the mass fraction of Cr_3_C_2_ reaches 30%, the overall hardness of the coating reaches its maximum at 825.4 HV3. Decarburization of Cr_3_C_2_ provides a source of carbon for the formation of multicomponent carbides of Fe, Ni, and other elements, resulting in the formation of ring-shaped phases (Ti, Cr, Fe, Ni)C around TiC particles that exhibit excellent wetting properties with the nickel-based binder phase.
3. The main wear mechanisms of the composite coating are abrasive wear and oxidative wear. With an increasing mass fraction of Cr_3_C_2_, both the friction and wear volume initially decreases and then increases, while the wear volume follows a similar trend. When the mass fraction of Cr_3_C_2_ reaches 26%, the friction coefficient is relatively low, the wear marks are narrower, and the wear volume is the lowest. However, at a mass fraction of 30% Cr_3_C_2_, the thermal stress increases, and the number of defects and cracks penetrating the coating surface increases, decreasing the wear resistance.

## Figures and Tables

**Table 1:** Chemical composition of Ni25A alloy powder.

Chemical Component	Cr	Fe	Ni	B	C	N	O	S	Si
Content (%)	7.530	2.590	balance	1.660	0.007	0.005	0.044	0.005	3.240

**Table 2:** Chemical composition of the coatings.

Samples	Binder Phase	Hard Phase (wt.%)	
	Ni25A	Cr_3_C_2_	TiC
1#	Balance	15.00	45.00
2#	Balance	19.00	45.00
3#	Balance	23.00	45.00
4#	Balance	26.00	45.00
5#	Balance	30.00	45.00

**Table 3:** Plasma surfacing process parameters.

Power (kW)	Current (A)	Overlap Butt Diameter (mm)	Scanning Speed (mm/s)	Powder Feeding Rate (g/min)	Atmosphere
17	120	4	14.0	75	Argon

**Table 4:** EDS analysis results of coating 1#.

Spot	Composition (at.%)					
	C	Fe	Ni	Ti	Cr	Si
Point A	45.37	13.94	6.81	25.53	9.83	0.35
Point B	51.51	4.87	0.38	39.35	3.05	0.52

**Table 5:** EDS results for coating 1#.

Spot	Composition (at.%)					
	Cr	Fe	Ni	Ti	C	Si
P1	0.78	0.42	0.21	48.02	51.01	/
P2	2.94	1.74	0.99	45.18	48.17	/
P3	4.52	13.17	10.14	36.06	34.99	0.38
P4	4.52	13.17	10.14	36.06	38.09	0.26
P5	8.15	40.53	27.65	1.5	20.57	1.6

**Table 6:** EDS analysis results of coating 1#.

Spot	Composition (at.%)				
	Cr	Fe	Ni	Ti	C
P6	3.05	4.87	0.38	39.35	51.51
P7	22.23	38.11	1.24	0.80	37.62

**Table 7:** Nanoindentation hardness results for coating 4#.

Indentation Point	1	2	3	4	5	6
Nanohardness (GPa)	32	23	20	16	6.6	5.1
Elastic modulus	368.16	344.62	283.91	256.87	274	225.35

**Table 8:** Coating wear rates of different samples.

Sample	Friction Stroke (m)	Wear Volume (mm^3^) × 10^−8^	Normal Force (N)	Wear Rate (mm^3^ N^−1^ m^−1^) × 10^−8^
1#	0.005	1.008	50	4.032
2#	0.005	1.012	50	4.048
3#	0.005	1.084	50	4.337
4#	0.005	0.938	50	3.750
5#	0.005	0.874	50	3.497

**Table 9:** EDS results of coating 1#.

Element	Cr	Fe	Ni	Ti	C	O
at%	7.33	4.06	13.07	15.91	26.53	29.56

## Data Availability

The data presented in this study are available on reasonable request from the corresponding author.
